# Does Urge To Drink Predict Relapse After Treatment?

**Published:** 1999

**Authors:** Damaris J. Rohsenow, Peter M. Monti

**Affiliations:** Damaris J. Rohsenow, Ph.D., is a research professor of community health and research director of the Addictive Behavior Research Laboratory at the Center for Alcohol and Addiction Studies, Brown University, and is a career research scientist at the Veterans Affairs Medical Center, Providence, Rhode Island. Peter M. Monti, Ph.D., is a professor of psychiatry and human behavior and associate director of the Center for Alcohol and Addiction Studies, Brown University, and is a senior career research scientist at the Veterans Affairs Medical Center, Providence, Rhode Island

**Keywords:** AOD (alcohol and other drug) craving, AODD (alcohol and other drug dependence) relapse, treatment outcome, AOD abstinence, scientific model, AOD withdrawal syndrome, motivation, social learning theory, CNS (central nervous system) information processing, alcohol cue, coping skills, literature review

## Abstract

The urge to drink, also often referred to as craving, is an emotional state in which a person is motivated to seek and use alcohol. In abstinent alcoholics, this urge may contribute to the risk of relapse. Researchers have developed several models—including the conditioned withdrawal model, conditioned appetitive motivational model, social learning model, and information-processing model—to describe the role of urges in relapse. Several studies have evaluated the role of urges in predicting alcoholism treatment outcome and relapse. Some findings indicate that the degree of urge an alcoholic experiences when confronted with a simulated high-risk situation at the end of alcoholism treatment can predict subsequent drinking. Other studies, however, show inconsistent results regarding the role of urges in predicting treatment outcome. Overall, the study results suggest that urges do not necessarily increase the risk of relapse but may actually protect some drinkers against further drinking.

Researchers and clinicians have long considered the urge to drink (also commonly called craving) a key cause of relapse following alcoholism treatment. Accordingly, craving has been a focus of many treatment approaches, a strong concern in some medication development programs, and a central aspect of various theoretical formulations of alcoholism and relapse. Some researchers, however, have raised doubts about the extent to which craving plays a role in drinking after treatment. Several factors contribute to the reevaluation of craving’s role in relapse. For example, some studies have demonstrated that alcohol consumption does not necessarily lead to loss of control over drinking among recovering alcoholics (Nathan and Lisman 1976). Furthermore, according to some laboratory studies, only moderate (although statistically significant) correlations exist between urges and alcohol consumption (Tiffany 1990). Finally, clinicians have noted that in many patients, relapse is not necessarily preceded by urges.

Few studies actually have investigated the role of craving in relapse among abstinent alcoholics. Consequently, investigations into the role of craving have become an important focus of many research programs that include not only alcoholics but also smokers and cocaine-dependent patients. Those analyses should help provide a better understanding of the role of urges in both treatment and treatment outcome (Monti et al. in press).

This article reviews some of the models used to explain the relationship between craving and relapse. It also presents some methods for assessing craving as well as the results of studies investigating the relationship between urges and relapse to drinking. Finally, the article discusses the implications of those results. Most of the theories presented here refer to drug abuse in general; the review of empirical studies, however, is limited to studies of alcoholics.

## Definition of Craving or the Urge to Drink

The terms “craving” and “urge to drink” often are used interchangeably in the literature. However, consistent with Kozlowski and colleagues (1989), the authors of this article prefer using the terms “urge to drink” and “desire to drink” in both research and practice, because the meaning of the term “craving” frequently is ambiguous or inconsistent. For example, many researchers, clinicians, and alcoholics use the term “craving” only to refer to an intense desire to drink, whereas others use the term to refer to urges with a wide range of intensities. Furthermore, researchers do not yet know whether even mild urges or only strong urges contribute to relapse. Consequently, investigators must study the effects of the entire range of motivations for drinking, a concept that is better served by the terminology “urge to drink.” Therefore, this terminology is used for the remainder of this article.

Urge to drink (also called “desire to drink,” “need to drink,” “want to drink,” and “missing drinking” in the treatment and research literature) is generally conceptualized as an emotional state that is characterized by the motivation to seek and use alcohol (Baker et al. 1987). This motivation, which can be associated with either positive or negative emotions (e.g., anticipation of alcohol’s positive effects or frustration over problems at work), is thought to be predictive of drinking.

### Characteristics of the UrgeTo Drink

Urges are inherently a self-reported phenomenon—that is, a person must in some way describe his or her desire to drink. Some researchers who work with animal models assess craving in terms of alcohol- or drug-seeking behavior (i.e., how much alcohol an animal drinks voluntarily). According to this approach, an animal that drinks more alcohol is considered to have a greater urge to drink. In humans—particularly in treatment populations—this approach is not appropriate, however, because a discrepancy exists between the urge to drink and actual alcohol consumption. This discrepancy results from the conflict between two competing motivations: (1) the motivation to drink (e.g., when thinking about alcohol’s pleasurable effects) and (2) the concurrent motivation not to drink (e.g., when recalling severe negative consequences of drinking). In fact, a recovering alcoholic’s ability to refrain from drinking despite urges to drink is of paramount interest to clinicians, and the strengthening of that ability is a key treatment focus.

Similarly, urges are not the same as psychophysiological reactions (e.g., changes in heart rate, blood pressure, skin conductance, and salivation) in situations that presumably elicit urges. Most of these responses are general measures of arousal, rather than specific indicators of urges, and therefore do not correlate well with urges.^1^ Salivation in response to images or objects associated with alcohol use (i.e., alcohol cues) probably reflects conditioned learning processes—that is, responses that are learned when two stimuli (one that is neutral and one that causes the response) are paired repeatedly. However, salivating in response to cues is also not the same as an urge, because people often are not aware of these reactions and thus do not report urges when salivating (Monti et al. 1993*b*). Therefore, whereas urges are inherently conscious processes, most psychophysiological changes are unconscious reactions and may reflect processes different from urges.

Urges also differ from intentions to drink or expectancies about the effects of drinking. Some recent studies have blurred the distinctions between those variables, assessing all three as though they were aspects of a single construct. Clinical observation, however, demonstrates that the three variables are distinct from each other, as follows:

- Alcoholics in treatment can expect that alcohol will have pleasurable effects but nonetheless still intend not to drink.
- Alcoholics can expect that alcohol will have negative effects (e.g., make them more depressed) and yet still intend to drink.
- Alcoholics can experience urges to drink without any intention to drink.

Again, researchers and clinicians are primarily interested in analyzing and enhancing these distinctions. The goal of their investigations is to identify situations in which abstinent alcoholics may have urges or positive expectations regarding alcohol’s effects but nevertheless still intend not to drink.

Some of the debate about the role of urges in drinking behavior may stem from insufficient distinctions being made between the role of urges in current drinkers as opposed to abstinent alcoholics, in whom urges may increase the risk for relapse. A current drinker may experience frequent strong urges as a result of recent drinking. In this situation, the ability of urges to predict future drinking independent of recent drinking is unclear. Alternatively, a current drinker may not experience more than a slight urge, because whenever the drinker feels any desire for a drink, he or she can have one with minimal delay. Conversely, urges in abstinent alcoholics may rise to high levels because drinking is prevented (either voluntarily or involuntarily). These urges may be particularly strong in situations that had been previously associated with drinking. This article investigates the ability of urges to predict subsequent drinking only in abstinent alcoholics, not in current drinkers.

## Models of the Role of Urges in Relapse

Formalized models of the role of urges can serve three major functions: (1) to shape current thinking about urges, (2) to help investigators develop research designs, and (3) to guide clinical choices. Numerous models have addressed urges, including the conditioned withdrawal model, the conditioned appetitive motivational model, the social learning model, and the information-processing model. The following sections briefly review these four models, all of which have been used to guide research. For more extensive discussions of the models and the evidence for and against them, readers are referred to other review articles (Niaura et al. 1988; Rohsenow et al. 1994). Because many predictions from these models are not mutually exclusive, researchers and clinicians cannot easily support or refute any model unequivocally.

### The Conditioned Withdrawal Model

This model suggests that when a person repeatedly undergoes withdrawal episodes, a conditioning process occurs. Through this process, the unpleasant symptoms of withdrawal (e.g., tremors, agitation, and anxiety) become associated with stimuli present during the withdrawal episodes (e.g., treatment settings, such as a hospital or therapist’s office) (see, for example, Ludwig et al. 1974). As a result, exposure to those stimuli will induce a mild withdrawal syndrome, which, in turn, will result in urges to drink to relieve withdrawal.

Research findings and clinical observation, however, do not provide strong support for this model. For example, patients usually experience few urges in treatment settings; in contrast, they experience strong urges in settings in which drinking has taken place previously. Furthermore, responses to withdrawal-associated stimuli do not consistently resemble withdrawal symptoms. For example, the model would predict that drug users should respond to the sight of opiates with increased arousal and to the sight of cocaine with decreased arousal (because opiate withdrawal causes increased arousal and cocaine withdrawal causes decreased arousal), yet users respond to both sets of cues with increased arousal (Rohsenow et al. 1990).

### The Conditioned Appetitive Motivational Model

In contrast to the conditioned withdrawal model, which suggests that urges develop to avoid the unpleasant consequences of withdrawal, the conditioned appetitive motivational model (see, for example, Stewart et al. 1984) proposes that urges result from the desire for alcohol’s pleasant effects. According to this model, emotional states characterized by the motivation to consume more alcohol (i.e., appetitive motivational states) become conditioned to stimuli (e.g., the sight of liquor bottles) and settings (e.g., bars) associated with the positive effects of drinking. Subsequent reexposure to the drinking-related stimuli is assumed to induce those appetitive motivational states as well as positive mood and increases in alcohol-related thoughts or urges to drink. Thus, both in this model and in the conditioned withdrawal model, urge accompanies learned reactions and may lead to drinking.

More experimental support exists for the conditioned appetitive motivational model than for the conditioned withdrawal model (Niaura et al. 1988). For example, reactions elicited in response to all types of drinking- or drug-related stimuli resemble the arousal associated with drug-seeking behavior (e.g., decreased skin resistance), whereas the conditioned withdrawal model would predict different reactions across various types of substances (Rohsenow et al. 1990). Furthermore the increased salivation that occurs when drinkers see alcohol resembles preparation for consumption (i.e., is an appetitive response).

### The Social Learning Model

According to the social learning model (see, for example, Abrams and Niaura 1987), relapse risk is associated with numerous learned factors. These factors include conditioned responses, positive expectancies about alcohol’s effects, ineffective coping responses, and the alcoholic’s expectation that he or she is unable to cope with a high-risk situation (i.e., a situation that is stressful or in which alcohol use has occurred previously) in any way other than drinking. The social learning model also posits that either conditioned stimuli or stressful situations can result in increased urges to drink, especially when combined with positive expectancies about alcohol’s effects. These urges, however, do not necessarily result in alcohol use, because other factors (e.g., expected negative consequences of drinking, the drinker’s ability to cope with stress in other ways, and the drinker’s belief in his or her own ability to handle the situation) affect a person’s decision or intention to drink. Thus, the social learning model considers urge as only one of many factors that can contribute to relapse and which is necessary but not sufficient for relapse to occur.

Many predictions made by the social learning model have been supported in experimental and clinical studies (Niaura et al. 1988). In fact, the social learning model has provided the most guidance to clinicians because of the wealth of variables (e.g., expectancies, conditioned responses, coping skills, and urges) that it incorporates. For example, coping-skills-training methods have been derived directly from this model (e.g., Monti et al. 1989).

### The Information-Processing Model

Instead of implicating conditioning processes, the information-processing model proposes that much alcohol-seeking behavior in alcoholics is controlled by automatic overlearned processes^2^ rather than by conscious thoughts (Tiffany 1990; also see the article in this issue by Tiffany, pp. 215–224). These automatic processes occur in the presence of stimuli associated with past drinking and may occur without awareness or without any urge on the part of the drinker. In contrast, urges result from conscious processes, such as problem-solving thoughts and increased attention or awareness, that are initiated when alcohol use is blocked, either involuntarily (e.g., when a drinker’s favorite bar is closed) or voluntarily (e.g., when an alcoholic decides to quit drinking).

According to the information-processing model, relapse can occur either through conscious processes, including urges, or through automatic drug-seeking processes and in the absence of urges. Thus, urges are not necessary for relapse to occur but, instead, may result from the person’s attempt to abstain from drinking in a high-risk situation. In that situation, urges reflect the desire to seek alcohol despite the conflicting wish to avoid alcohol use. Some experimental support exists for this model (Rohsenow et al. 1994), which is discussed in the section “Urges as Predictors of Outcome,” p. 229.

## Assessing Urges

When assessing the role of urges in relapse, researchers must consider the context in which alcoholics are queried about their urges to drink. For example, alcoholics undergoing treatment tend to experience only infrequent and mild urges, possibly because they are not exposed to alcohol-related stimuli that lead to urges. Consequently, urges reported during treatment may not predict drinking outcomes (Monti et al. in press). Furthermore, a patient’s risk of relapse likely is not constant but varies across several days or even over the course of a given day. In addition, certain situations or events—such as an argument with a family member, a celebration, or seeing friends drink at a party—may be more likely to provoke a relapse and thus represent an “Achilles’ heel” for the recovering alcoholic. Consequently, to obtain meaningful information about the relationship between urges and relapse risk, researchers should assess urges in contexts designed to provoke responses similar to those that may be experienced in real-life, high-risk situations. Two methods frequently used in urge assessment are role play and cue reactivity.

Role-play assessment presents alcoholics with 8 to 10 scenarios describing situations that pose a high risk for relapse among alcoholics—for example, feeling angry and frustrated after being unemployed for 1 month and yet being ineligible for unemployment benefits (Monti et al. 1993*a*). After each situation is presented, the alcoholics describe how they would handle the situation. Trained judges later evaluate those responses for the degree of coping skills exhibited. The alcoholics also rate (on a scale from 0 to 10) their urge to drink and their confidence that they can handle such a situation without drinking. One role-play assessment measure used in prediction studies is the Alcohol-Specific Role Play Test (ASRPT) (Monti et al. 1990).

Cue-reactivity assessment evaluates urges while asking alcoholics to hold and smell a glass of the alcoholic beverage that they most often drink. This type of evaluation mimics real-life situations in which alcoholics are exposed to alcohol but know that they must not drink it. In addition to rating their urge to drink, the patients rate the amount of attention they pay to the sight and smell of the drink and to their thoughts about alcohol as well as their sensory awareness (e.g., salivation or heart pounding). In addition, the investigator collects information on salivation and other psychophysiological measures (e.g., heart rate and skin conductance).

Both role-play and cue-reactivity assessment have excellent reliability and validity.^3^ For example, the urge ratings in the role-play test have good internal consistency and validity and have exhibited no gender differences in three samples of alcoholics (Monti et al. 1993*a*). Furthermore, the urge ratings in cue-reactivity assessment are highly reliable across repeated trials (e.g., Monti et al. 1993*b*). Also, some studies found that alcoholics reacted more strongly to alcohol-related cues than did nonalcoholics (Monti et al. 1987). Approximately 65 percent of the alcoholics experienced an increased urge to drink and approximately 70 percent exhibited increased salivation when exposed to alcohol cues (Rohsenow et al. 1992, 1994). In addition, other studies have demonstrated, as expected, that the urge to drink is largely independent of salivation and other psychophysiological measures (Niaura et al. 1988; Rohsenow et al. 1992).

Patients’ responses during cue-reactivity or role-play assessment have been found to correlate with various concurrent behaviors relevant to relapse risk, thereby supporting the validity of these assessment approaches. For example, urge in response to cues or to role-play situations is greater among alcoholics with greater alcohol dependence than among alcoholics with lesser alcohol dependence (Monti et al. 1993*b*; Rohsenow et al. 1992).

Some of the relationships between urges and other measures also suggest ways through which these reactions may increase relapse risk. For example, alcoholics who reacted early in treatment to alcohol cues with a greater urge to drink also performed less skillfully in the ASRPT during treatment, suggesting that reactions to alcohol cues disrupt the ability to use coping skills in high-risk situations (Monti et al. 1993*a*). In another study, alcoholics were asked to push a button in response to hearing a sound while they were simultaneously exposed to either alcohol-related or alcohol-unrelated cues (Sayette et al. 1994). In that study, the alcoholics’ reaction times for pushing the button were significantly longer when they were exposed to alcohol-related cues than when they were exposed to non-alcohol-related cues. These findings indicate that the presence of alcohol cues slows information processing by diverting the alcoholics’ attention. Both the study by Monti and colleagues (1993*a*) and the study by Sayette and colleagues (1994) suggest mechanisms through which stronger reactions to cues may place alcoholics at greater risk for relapse.

## Urges as Predictors of Outcome

Several studies using a variety of methods have assessed the role of urges in predicting treatment outcome and relapse. For example, Marlatt and Gordon (1985) asked alcoholics retrospectively about the situations in which they had relapsed and reported that urge was rarely the primary cause of relapse in those situations. The ability of such an approach to determine the role of urges in relapse is limited, however. For example, memory becomes less reliable and more selective over time, making retrospective information increasingly inaccurate. Also, Marlatt and Gordon (1985) likely underrepresented the importance of urges, because the researchers categorized the relapse situations as resulting from urges or craving only if no additional possible causes (e.g., conflicts with other people) occurred. Consequently, both laboratory studies and ambulatory monitoring techniques (e.g., use of small portable computers into which participants enter information immediately after a situation occurs) offer advantages over retrospective accounts for assessing the relevance of urges.

Other researchers have used a prospective approach to elucidate the role of urges. For example, Miller and colleagues (1996) asked alcoholics to rate their “urges” and “cravings” 4 months after treatment.^4^ The investigators then used this information to predict relapse or resumption of drinking after at least 4 days of abstinence during the next 2 months. The study found that the frequency of reported urges, but not the frequency of cravings, significantly predicted resumption of drinking during the subsequent 2-month period. One shortcoming of this study, however, was that not all participants were abstinent at the time the urges were assessed. Consequently, if drinking results in increased urges, the results may be somewhat tautological.

One study used the urge to drink and other responses to the ASRPT at the end of residential treatment to predict drinking during a 6-month followup period (Monti et al. 1990). Using statistical methods, the various self-report measures (i.e., urge to drink, anxiety, difficulty coping without drinking, and self-rated skill in coping) then were averaged across the situations presented during the ASRPT. These analyses found that urge to drink alone significantly predicted drinking quantity over the followup period, accounting for 26 percent of the variance in the alcohol amount consumed. The urge to drink induced during a procedure designed to produce stress similarly predicted drinking quantity during followup. The strongest predictor of subsequent drinking, however, was the urge to drink that persisted through the 3-minute recovery period after each role-play test. This variable accounted for 58 percent of the variance in drinking quantity and 41 percent of the variance in drinking frequency during the followup period. These findings indicate that the degree of urge at the end of treatment as assessed in a role-play test using simulated high-risk situations can predict subsequent drinking. Furthermore, lasting urges that persist even after the high-risk situation is over may be particularly predictive of outcome.

### Urges During Cue-Reactivity Assessment as Predictors of Outcome

Several studies evaluating the role of urges in response to alcohol beverage cues as predictors of drinking outcome have generated somewhat mixed results. Those analyses have allowed researchers to test some of the predictions from the information-processing model of relapse. According to that model, urges to drink account for only some of the variance in drinking after treatment, whereas additional variance may be associated with other unconscious and conscious processes. Unconscious automatic processes may be reflected by psychophysiological reactions, such as increased salivation or heart rate.

A conscious process that may influence the risk of relapse in addition to urges is the drinker’s attention in the presence of alcohol cues. The information-processing model suggests that attempts to avoid drinking in the presence of alcohol cues can increase a drinker’s attention to those cues and to his or her reactions to the cues (Tiffany 1990). This hypothesis regarding the role of attention is extended by social learning models suggesting that awareness of danger or of one’s own reactions is central to self-regulation of behavior (Bandura 1977) and may be crucial in mobilizing coping responses (Rohsenow et al. 1994).

Four cue-exposure studies have investigated the ability of automatic processes (e.g., salivation) and nonautomatic processes, such as urges and attention, to predict outcome after treatment, as follows:

- Monti and colleagues (1993*c*) used cue-elicited urges and salivation in the first few days of treatment to predict outcome in a small sample of alcoholics undergoing treatment in a Veterans’ Affairs (VA) facility. In that study, higher urge ratings predicted less frequent drinking. Furthermore, salivation did not predict drinking.^5^
- Drummond and Glautier (1994) also found that higher urge ratings at the end of treatment predicted a longer interval before relapse to heavy drinking. Conversely, psychophysiological arousal during that session predicted more rapid relapse.
- Cooney and colleagues (1997) noted that the urge to drink after exposure only to alcohol cues was not predictive of outcome. Instead, increased desire to drink predicted a shorter time to the first drink only among alcoholics who were exposed both to alcohol cues and to a procedure designed to induce negative mood.
- Rohsenow and colleagues (1994) studied a large group of alcoholics being treated at a VA facility to determine the ability of urge to drink, salivation, and self-reported attentional variables in the first few days of treatment to predict drinking outcomes (e.g., frequency of drinking during followup). In that study, urge to drink did not predict outcome. However, salivation was associated with treatment outcome, in that participants who salivated more drank more often during followup. In addition, the amount of attention the alcoholics reported paying to either the alcohol cues or to their sensory reactions to the cues significantly predicted outcome. Thus, alcoholics who reported paying more attention to either the cues or to their reactions to the cues drank significantly less during the followup period than did alcoholics who reported paying less attention.

The ability of both salivary reactions to alcohol cues and attentional variables to predict outcome is consistent with the information-processing model. Thus, salivation may reflect the automatic learning processes related to drinking (Rohsenow et al. 1994), whereas attention is considered an aspect of nonautomatic thought processes and is believed to correlate with attempts to inhibit desires to seek drugs. Accordingly, people who pay greater attention to alcohol cues probably inhibit their desire to seek alcohol, possibly because greater attention to high-risk situations or to one’s reactions to those situations facilitates mobilization of coping skills, thereby helping to handle the situation more effectively (Rohsenow et al. 1994). This greater use of coping skills, in turn, predicts less drinking. Conversely, alcoholics who pay less attention to alcohol cues or to their own reactions may be less aware of the riskiness of these situations and therefore may be controlled to a greater extent by their automatic overlearned behaviors (as indicated by increased salivation). Through this mechanism, lack of attention may increase a recovering alcoholic’s likelihood of drinking in high-risk situations.

In contrast to salivation and attentional variables, the results regarding the role of urge in drinking outcome were inconsistent, with urge predicting less drinking in some studies and more drinking in others. Thus, although a positive correlation exists between urge to drink and attention to alcohol cues (i.e., the greater a drinker’s urge, the more attention he or she will pay to alcohol cues) (Monti et al. in press; Rohsenow et al. 1994), the two variables differ in their ability to predict outcome.

One explanation for this inconsistency may be that newly abstinent alcoholics who are in treatment generally experience conflict: On the one hand, they are motivated to drink to experience alcohol’s effects; on the other hand, they are motivated not to drink for a variety of reasons. As a result of this conflict, the urge to drink among newly abstinent alcoholics may consist of two components: (1) motivation to drink and (2) awareness of the danger of relapse. The variance in urge to drink that results from awareness of danger should induce the alcoholic to initiate coping skills that reduce the risk of drinking. However, some situations are easier to cope with than others. For example, many alcoholics report little difficulty refusing a drink during coping-skills training (e.g., Monti et al. 1989). This finding may explain why the urge to drink during alcohol cue exposure may be unrelated to future drinking or may even protect against later drinking. Complex emotional situations (e.g., problems at work or conflicts with family members), in contrast, may be more difficult to cope with effectively. Thus, studies using negative mood induction or the complex social situations represented in the ASRPT found that the urge to drink in those situations predicted later drinking. (Cooney et al. 1997; Monti et al. 1990). According to the information-processing and social learning models, those complex situations may overwhelm an alcoholic’s coping resources or the nonautomatic mental efforts needed to prevent drug-seeking behavior. As a result, more intense urges to drink in these complex situations increase the likelihood of drinking.

## Clinical Implications

The research findings regarding the role of urges described in the previous section have several clinical implications. Most important, the results indicate that urges may not necessarily increase the risk of drinking but may actually protect against drinking under certain circumstances (e.g., in drinkers who are aware of alcohol cues and of their reactions to those cues). Many alcoholics try to prevent themselves from experiencing urges, possibly because they consider them to be a risk for relapse. As a result, however, some of those alcoholics may develop an overconfidence in their ability to cope with high-risk situations or may underestimate the riskiness of some situations.

Monti and colleagues (1993*b*) found that alcoholics who salivated relatively more in response to alcohol cues (i.e., who were at greater risk for drinking) only reported an increased urge to drink if they also were aware of salivating more. These findings suggest that the experience of urges may require some self-awareness. Consequently, for alcoholics in high-risk situations who may experience psychophysiological reactions, an increased awareness of their reactions in such situations (including the urge to drink) and of the potential danger of relapse may protect them against drinking by prompting them to mobilize their coping resources. In highly complex situations that pose too great a challenge for coping, however, urges may signal an increased risk of relapse.

Within this conceptualization of the role of urges in the risk of relapse, several factors appear to increase an alcoholic’s probability of drinking when experiencing the urge to drink (Monti et al. in press):

- Factors that increase the motivation to drink, such as positive expectancies about alcohol, negative emotions, and certain physiological states (e.g., low levels of certain chemicals in the brain)
- Factors that decrease the awareness of danger, such as overconfidence or maladaptive beliefs about the riskiness of a situation as well as physiological states that decrease general awareness (e.g., overtiredness)
- Factors that decrease the effectiveness of coping, such as inadequate coping skills, highly complex situations, failure to believe in one’s ability to cope effectively, and physiological states that impair cognitive abilities (e.g., exhaustion or illness).

Clinicians can help alcoholics recognize that urges are a danger sign that should not be ignored but should be considered an indication that coping resources must be mobilized. Furthermore, certain alcoholism treatment approaches, such as coping-skills training, can strengthen an alcoholic’s ability to cope with urges and risky situations as well as increase confidence in his or her own ability to apply coping skills (Monti et al. 1989). Certain urge-specific coping skills in particular—such as coping with urges by thinking about the positive consequences of staying sober and the negative consequences of drinking—are associated with less frequent drinking after treatment (Monti et al. 1993*c*).

## Future Directions

Investigators are currently pursuing several lines of research to further clarify the role of urges in recovery after alcoholism treatment. For example, some researchers are investigating the effects of naltrexone—a medication that was approved for alcoholism treatment in 1994—on the urge to drink as well as the relationship between these effects and outcome (i.e., amount and frequency of drinking). In one controlled laboratory study, naltrexone reduced the number of alcoholics who experienced any urge to drink; however, the medication did not affect the magnitude of the urges to drink among those alcoholics who reported experiencing such urges (Monti et al. in press). Researchers currently do not know whether urge reduction is the primary mechanism through which naltrexone improves drinking outcome, but that issue is under investigation.

A second interesting line of research involves the extent to which changes in cue-elicited urges are associated with changes in the levels of a certain brain chemical (i.e., the neurotransmitter dopamine) in the brain regions that mediate the rewarding effects of alcohol (Monti et al. in press). Many researchers think that higher dopamine levels in those brain regions are related to greater urges, and that medications which reduce dopamine levels in those areas could prevent urges and thus be useful in alcoholism treatment.

In a third line of research, investigators are assessing the effects of extended cue-exposure treatment (both by itself and in combination with either training for coping with urges or with communication skills training) on drinking outcome. This treatment approach was piloted in a small study that suggested such combinations of cue-exposure treatment with coping-skills training could improve treatment outcome (Monti et al. 1993*c*). Another study suggested that cue-exposure treatment even without any skills training could reduce future drinking (Drummond et al. 1994). The results of a subsequent larger trial will be released soon.

In conclusion, the urge to drink among recovering alcoholics is a construct of considerable interest to researchers as well as clinicians that is finally being rigorously studied. The studies reviewed in this article demonstrate that even inherently subjective variables, such as the urge to drink, can contribute considerably to the understanding of the factors that determine relapse and recovery when measured with methodological rigor and in meaningful contexts. Such analyses can be heuristic in guiding clinical practice.

## Footnotes

1 This lack of correlation can be expected if the information-processing theory of urges, which is discussed later in this article, is valid.

2 Automatic overlearned processes are behaviors that are repeated so frequently that they are performed with little awareness and effort (e.g., eating, dressing, or driving a car).

3 Reliability means that if the same person is tested several times, the results will be consistent across the tests (test-retest reliability) or if a concept (such as urge) is tested using a number of items, the person will respond consistently across the items (internal consistency). Validity means that the test has been shown to measure effectively the variable it is supposed to measure.

4 The terms “urges” and “cravings” were not specifically defined in that study and their meaning therefore may not correspond to the definitions used in the present article.

5 Because the study used only a small sample, its statistical power to detect the effects of salivation was limited.
